# Association of the COVID-19 Pandemic With Estimated Life Expectancy by Race/Ethnicity in the United States, 2020

**DOI:** 10.1001/jamanetworkopen.2021.14520

**Published:** 2021-06-24

**Authors:** Theresa Andrasfay, Noreen Goldman

**Affiliations:** 1 Leonard Davis School of Gerontology, University of Southern California, Los Angeles; 2 Office of Population Research and Princeton School of Public and International Affairs, Princeton University, Princeton, New Jersey

## Abstract

This cross-sectional study examines the association of the COVID-19 pandemic with estimated life expectancy by race/ethnicity in the US.

## Introduction

In October 2020, we estimated the impact of COVID-19 on 2020 period life expectancy at birth in the US by race/ethnicity using observed COVID-19 deaths from February 1, 2020, to October 3, 2020, and projected COVID-19 deaths through the remainder of 2020 under 3 scenarios (low, medium, and high COVID-19 mortality).^1^ Under the medium scenario, we projected a decline in 2020 US life expectancy of 1.13 years for the total population, 0.68 years for the White population, 2.10 years for the Black population, and 3.05 years for the Latino population.^1^ This study updates these estimates with observed deaths for all of 2020 and more recent prepandemic mortality conditions.

## Methods

This cross-sectional study uses publicly available data and was exempt from institutional review board approval and informed consent, per the institutional review board of Princeton University. This study follows Strengthening the Reporting of Observational Studies in Epidemiology (STROBE) reporting guideline for cross-sectional studies; the supplementary material provides additional details.

This cross-sectional study uses provisional COVID-19 death counts by week, race/ethnicity, and age from the Centers for Disease Control and Prevention’s National Center for Health Statistics (NCHS); 2019 mid-year population estimates by race/ethnicity and age from the US Census Bureau; and 2018 US life tables by race/ethnicity from the US Vital Statistics System. We treat 2018 life tables as counterfactuals to 2020 life tables in which COVID-19 has been eliminated and estimate 2020 life expectancy including COVID-19 mortality (eMethods in the Supplement). Race/ethnicity was included to estimate disparities in life expectancy reductions. In census data, race/ethnicity is self-reported; in the mortality data, race/ethnicity is typically recorded by funeral directors with information from the decedent’s next of kin. To estimate life expectancy reductions in 2021 thus far, we repeat these calculations using provisional deaths through early April 2021. Because we include all reported COVID-19 deaths, we do not test for significant differences.

## Results

The calculations of life expectancy include 380 868 COVID-19 deaths in 2020. Race/ethnicity data were available for more than 99% of these deaths, including 230 016 among non-Latino White individuals, 60 405 among non-Latino Black individuals, and 69 066 among Latino individuals. Updated estimates indicate that COVID-19 reduced overall 2020 US life expectancy by 1.31 years, from 78.74 years to 77.43 years (Table and Figure). The reductions are 3.2 times as large for the Latino population (3.03 years) and twice as large for the Black population (1.90 years) compared with the White population (0.94 years).

**Table.** Updated and Previous Estimates of 2020 US Life Expectancy at Birth by Race and Ethnicity

	Population			
	Total	Non-Latino White	Non-Latino Black	Latino
Updated 2020 estimates				
COVID-19 deaths, No.^a^	380 868	230 016	60 405	69 066
Absent COVID-19 life expectancy (2018 Mortality), y	78.74	78.63	74.71	81.83
Estimated 2020 life expectancy, y	77.43	77.69	72.81	78.80
Reduction in life expectancy, y	−1.31	−0.94	−1.90	−3.03
Previous 2020 estimates (medium scenario)^b^				
COVID-19 deaths, No.	321 140	166 947	67 050	69 057
Absent COVID-19 life expectancy (2017 Mortality), y	78.61	78.52	74.88	81.82
Estimated 2020 life expectancy, y	77.48	77.84	72.78	78.77
Reduction in life expectancy, y	−1.13	−0.68	−2.10	−3.05
Preliminary 2021 Estimates				
COVID-19 deaths through April 4, 2021, No.^c^	158 854	98 243	18 519	31 652
Reduction in life expectancy, y	−0.58	−0.44	−0.62	−1.47

^a^ Provisional COVID-19 death counts include all deaths for which the underlying cause or a contributing cause of death is *International Statistical Classification of Diseases and Related Health Problems, Tenth Revision* (*ICD-10*) code U07.1 (*ICD-10* code for COVID-19), regardless of whether the infection was laboratory-confirmed.

^b^ Previous 2020 estimates refer to the life expectancy estimates made in Andrasfay and Goldman^1^ based on projected deaths from October through December 2020.

^c^ Deaths from January 1, 2021 through April 4, 2021 are obtained from the April 7, 2021, National Center for Health Statistics release of provisional weekly COVID-19 deaths; due to delays in reporting, these totals differ from other sources.

**Figure.**
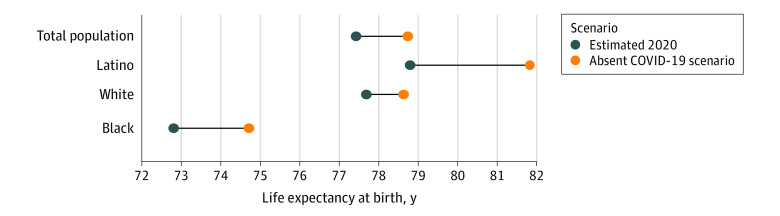
Updated Estimates of 2020 US Life Expectancy at Birth by Race and Ethnicity Estimates for 2020 life expectancy were calculated using COVID-19 deaths reported to the National Center for Health Statistics. The absent COVID-19 scenario is assumed to be the mortality conditions of 2018.

## Discussion

This cross-sectional study found that changes in life expectancy associated with COVID-19 for the total US and White populations exceeded those previously estimated.^1^ The change in life expectancy among the Black population was smaller than projected but remained much larger than that for the White population. The differences are likely partly due to the changing geography of the outbreak: since our October projections, Midwestern and Mountain states with large White populations experienced surges in COVID-19 cases and deaths. A minor change in estimates resulted from using 2018 rather than 2017 for the absent COVID-19 scenario.

Despite a prepandemic 7-year advantage in life expectancy compared with the Black population and 3-year advantage compared with the White population, along with lower rates of smoking and most chronic conditions,^2^ the Latino population had the largest life expectancy decline associated with COVID-19. This unprecedented change likely stems from social and economic inequities that are associated with both higher exposure to infection and higher fatality among those infected. Compared with Black and White individuals, Latino individuals in the US have lower rates of health insurance (affecting access to testing, treatment, and quality health care),^2,3^ are more apt to live in multigenerational and crowded households,^3,4^ and are more likely to hold frontline jobs involving risks of viral transmission without adequate protection.^3,5^ Language barriers may prevent Latino individuals from receiving timely, comprehensible, and accurate information regarding COVID-19 or effective strategies to protect themselves.^3,4^ High poverty rates and low status jobs offer Latino individuals few avenues for reducing viral exposure in the absence of government programs.^3,5^

A limitation of this study is that these life expectancy reductions are almost certainly underestimated because they exclude misclassified COVID-19 deaths and mortality from causes resulting indirectly from COVID-19. Recent estimates suggest that such underestimates may be especially large for the Black population^6^ and thus likely affect racial disparities in life expectancy.

The findings of this cross-sectional study suggest that, consistent with our earlier speculation,^1^ COVID-19 is associated with continued reductions in life expectancy in 2021 compared with prepandemic levels. COVID-19 deaths through early April 2021 already indicate an almost 0.6-year reduction in overall 2021 US life expectancy with continued disproportionate changes for the Black and Latino populations.

## Notes

Supplement. eMethods.
