# Supplementary material for: Association of the COVID-19 Pandemic With Estimated Life Expectancy by Race/Ethnicity in the United States, 2020
Source: JAMA Netw Open. 2021 Jun 24;4(6):e2114520. doi: 10.1001/jamanetworkopen.2021.14520 (PMC8226419; doi:10.1001/jamanetworkopen.2021.14520)
Supplement: Supplement. — eMethods. [file jamanetwopen-e2114520-s001.pdf]

Supplemental Online Content

Andrasfay T, Goldman N. Association of the COVID-19 pandemic with estimated life expectancy by race/ethnicity in the United States, 2020. *JAMA Netw Open*. 2021;4(6):e2114520. doi:10.1001/jamanetworkopen.2021.14520

eMethods

This supplemental material has been provided by the authors to give readers additional information about their work.

© 2021 Andrasfay T et al. *JAMA Network Open.*

eMethods.
Data Sources

COVID-19 death counts by week, race, Hispanic origin[a] and age group are obtained from the National Center for Health Statistics (NCHS).[1] These are provisional counts from death certificates reported to NCHS that have not been adjusted for delays in reporting or underreporting. At the time of writing, this demographic information is available for COVID-19 deaths occurring in each week from the week beginning December 29, 2019 through the week ending April 4, 2021, though NCHS cautions that data for recent weeks are incomplete because of delays in reporting to NCHS. These counts include all deaths for which the underlying cause or a contributing cause of death is ICD-10 code U07.1 (ICD-10 code for COVID-19), regardless of whether the infection was laboratory-confirmed.

Mid-year population counts by race, Hispanic origin, and age are obtained from the US Census Bureau's estimates for 2019, the most recent year for which these population counts are available.[2] We assume that the 2020 population distribution by race, ethnicity, and age is equivalent to the 2019 population distribution.

Estimates of mortality rates and other life table quantities by race, Hispanic origin, and age are taken from the US life tables published by the National Vital Statistics System for the year 2018, the most recent year for which these life tables are available.[3] We assume that, in the absence of COVID-19, mortality conditions in 2020 would be equivalent to those observed in 2018.

Methods

The total number of deaths from COVID-19 in 2020 in the United States by race, ethnicity, and age group are calculated as the total of all COVID-19 deaths from the week beginning December 29, 2019 to the week beginning December 27, 2020. Because the final week of 2020 includes deaths through January 2, 2021, we subtract two-sevenths of the deaths in this week and add them to the first full week in 2021. This results in a total of 380,868 COVID-19 deaths in 2020 and 158,854 COVID-19 deaths in 2021 from January 1, 2021, through April 4, 2021.

We then treat the 2018 life tables as cause-deleted life tables in which COVID-19 is the cause that has been deleted and then recover the all-cause life tables that include deaths from COVID-19. A cause-deleted life table, also known as an associated single decrement life table, is a hypothetical set of age-specific mortality rates and other life table quantities that would be expected if a single cause of death were eliminated.[4]

To do so, we do the following intermediate calculations for each racial and ethnic group:

1. Calculate estimated number of deaths in 2020 in the absence of COVID-19 (${}_nD_x{}^*$)[b] by applying the 2018 age-specific mortality rates (${}_nM_x{}^{18}$) to the 2019 population count by age interval (${}_nK_x{}^{19}$).

$${}_nD_x{}^* = {}_nM_x{}^{18} * {}_nK_x{}^{19}$$

2. Calculate estimated number of deaths in 2020 with COVID-19 by applying the 2018 age-specific mortality rates (${}_nM_x{}^{18}$) to the 2019 population count by age interval (${}_nK_x{}^{19}$), less the number who died from COVID-19 in this age group. Then we add in the total COVID-19 deaths by age group.

© 2021 Andrasfay T et al. *JAMA Network Open.*

$$ {}_nD_x^{20} = {}_nM_x^{18} * \left[{}_nK_x^{19} - {}_nD_x^{COV} \right] + {}_nD_x^{COV} $$

where ${}_nD_x^{20}$ is the estimated number of deaths between ages x and x + n in 2020 and ${}_nD_x^{COV}$ is the estimated number of deaths between ages x and x + n from COVID-19.

3. Calculate the age-specific ratio of deaths in the absence of COVID-19 to deaths in the presence of COVID-19. This is equivalent to the ratio of deaths from a single cause (in this case deaths from all but one cause) to deaths from all causes (${}_nR_x$) from Chiang's method.[5]

$$ {}_nR_x = \frac{{}_nD_x^{*}}{{}_nD_x^{20}} $$

4. Calculate the inverse of ${}_nR_x$, which simplifies some later calculations and can be interpreted as the ratio of deaths or mortality rates in the presence of COVID-19 to deaths or mortality rates in the absence of COVID-19.
5. Using Chiang's method, which assumes that the force of decrement from cause *i* (or, alternatively, all causes except *i*) is proportional to the force of decrement from all other causes, we can calculate the probability of surviving between ages x and x+n (${}_np_x^{20}$), the probability of dying between ages x and x+n (${}_nq_x^{20}$), and the average number of person-years lived by those who die between ages x and x+n (${}_na_x^{20}$) in the presence of COVID-19:

$$ {}_np_x^{20} = e^{\left(\frac{1}{{}_nR_x} * \log({}_np_x^{18})\right)} $$

$$ {}_nq_x^{20} = 1 - {}_np_x^{20} $$

$$ {}_na_x^{20} = n + \frac{1}{{}_nR_x} * \frac{{}_nq_x^{18}}{{}_nq_x^{20}} * \left({}_na_x^{18} - n \right) $$

$$ {}_\infty a_{85}^{20} = {}_\infty a_{85}^{18} * {}_\infty R_{85} $$

We then complete the rest of the life table for 2020 in the presence of COVID-19 using standard life table relationships.[4] This process is separately repeated for all racial and ethnic groups. Last, we repeat this process for 2021 using provisional deaths through April 4, 2021.

---

[a] In the text, we use the term "Latino" to refer to individuals with Latino or Hispanic origin. In this supplementary material, we use the term from the original source.

[b] In demographic notation the subscript x refers to exact age x, n refers to the length of the age interval. ${}_nD_x$ is interpreted as the number of deaths (D) between ages x and x + n.

© 2021 Andrasfay T et al. *JAMA Network Open.*

References

1. National Center for Health Statistics. Provisional Weekly Deaths by Region, Race, Age. Published online April 7, 2021. Accessed April 8, 2021. https://data.cdc.gov/NCHS/Provisional-Weekly-Deaths-by-Region-Race-Age/tpcp-uiv5

2. U.S. Census Bureau, Population Division. Data from “Annual Estimates of the Resident Population by Sex, Age, Race, and Hispanic Origin for the United States: April 1, 2010 to July 1, 2019.” Published online June 25, 2020. Accessed July 7, 2020. https://www.census.gov/newsroom/press-kits/2020/population-estimates-detailed.html

3. Arias E, Xu J. United States Life Tables, 2018. Published online November 17, 2020. Accessed January 6, 2021. https://www.cdc.gov/nchs/data/nvsr/nvsr69/nvsr69-12-508.pdf

4. Preston S, Heuveline P, Guillot M. *Demography: Measuring and Modeling Population Processes. 2001.*; 2000.

5. Chiang CL. The life table and its construction. *Introduction to Stochastic Processes in Biostatistics*. Published online 1968:198-214.

© 2021 Andrasfay T et al. *JAMA Netw Open.*
